# Primitive ATP-activated P2X receptors: discovery, function and pharmacology

**DOI:** 10.3389/fncel.2013.00247

**Published:** 2013-12-06

**Authors:** Samuel J. Fountain

**Affiliations:** School of Biological Sciences, University of East AngliaNorwich, UK

**Keywords:** P2X receptor, evolution, molecular, pharmacology, structure-activity relationship, ion channels

## Abstract

Adenosine 5-triphosphate (ATP) is omnipresent in biology. It is therefore no surprise that organisms have evolved multifaceted roles for ATP, exploiting its abundance and restriction of passive diffusion across biological membranes. A striking role is the emergence of ATP as a *bona fide* transmitter molecule, whereby the movement of ATP across membranes serves as a chemical message through a direct ligand-receptor interaction. P2X receptors are ligand-gated ion channels that mediate fast responses to the transmitter ATP in mammalian cells including central and sensory neurons, vascular smooth muscle, endothelium, and leukocytes. Molecular cloning of P2X receptors and our understanding of structure-function relationships has provided sequence information with which to query an exponentially expanding wealth of genome sequence information including protist, early animal and human pathogen genomes. P2X receptors have now been cloned and characterized from a number of simple organisms. Such work has led to surprising new cellular roles for the P2X receptors family and an unusual phylogeny, with organisms such as *Drosophila* and *C. elegans* notably lacking P2X receptors despite retaining ionotropic receptors for other common transmitters that are present in mammals. This review will summarize current work on the evolutionary biology of P2X receptors and ATP as a signaling molecule, discuss what can be drawn from such studies when considering the action of ATP in higher animals and plants, and outline how simple organisms may be exploited experimentally to inform P2X receptor function in a wider context.

## INTRODUCTION

Seminal early discoveries identified the role of acetylcholine and norepinephrine in chemical transmission at neuronal synapse. However, the concept of non-adrenergic non-cholinergic (NANC) chemical transmission mediated by adenosine 5-triphosphate (ATP) emerged much later and was met initially with sizable resistance amongst the pharmacology and neuroscience communities (Burnstock, 2012). The development of the hypothesis that ATP can act as a transmitter molecule also came to challenge “Dales Principle” which suggested that a neuron only utilizes one transmitter molecule to communicate. It is now well accepted that neurons of the central and peripheral nervous systems release ATP as a co-transmitter. Indeed ATP is the sole transmitter molecule to act post-synaptically in some instances, for example during sympathetic innervation of submucosal arterioles of the intestine (Evans and Surprenant, 1992). Some molecules that are associated with neurotransmission such as GABA and glutamate are utilized by primitive organisms, which lack nervous systems, for the purpose of cellular signaling (Fountain, 2010).

The human genome encodes a family of cell surface receptors that are activated by extracellular adenine and uridine signaling nucleotides. The metabotropic arm of this family are the G protein-coupled P2Y (mammalian P2Y_1,2,4,6,11,12,13,14_) receptors that respond to ATP, ADP, UTP, UDP, and UDP-glucose with receptor subtype selectivity, and typically mediate slow responses to nucleotides. P2X receptors (mammalian P2X_1__-__7_) are ligand-gated ion channels and mediate fast responses solely to ATP. In mammals, P2X receptor activation is associated with diverse physiological and pathophysiological processes including pain, inflammation, taste and smooth muscle contraction (Khakh and North, 2006). P2X receptors are non-selective cation channels, consequently receptor activation leads to membrane depolarization and cellular calcium influx, both by direct receptor permeation of calcium and voltage-gated calcium channel activation (North, 2002).

Adenosine 5-triphosphate is omnipresent in biology and plays many important roles including energy transfer and as a phosphate donor in enzymatic reactions, though its acceptance as a certified signaling ligand was slow despite early reports of potent physiological action in mammalian systems. The effects of exogenous ATP on insects and invertebrates has been known for some time (for a review see Burnstock and Verkhratsky, 2009), though the identification and cloning of P2X receptors from invertebrates and primitive single-celled organisms has been far less widespread. The identification of genes encoding putative P2X receptors in primitive organisms has been aided by a wealth of structure-function information for mammalian receptors, and an ever-expanding library of curated genomes for single-celled organisms including algae, amoeba and basal fungi. For example, the P2X_A_ receptor of *Dictyostelium discoideum* shares only very low primary sequence homology with mammalian P2X receptors (Fountain et al., 2007). A translated BLAST search using the full-length 378 amino acid sequence of the *Dictyostelium* as a query, provide no homologous mammalian P2X receptor sequences when using an expect value (*E* value) of 10. However, using the second transmembrane (TM2) domain as a search term returns hits of mammalian homologous, giving confidence of authenticity based on the conserved pattern of TM2 residues known to be critical to the function of mammalian P2X receptors. Bioinformatics is a powerful approach for expanding the phylogeny of primitive P2X receptors, however, caution must be applied when making structural or functional inferences to mammalian P2X orthologues without cloning and definitive demonstration of functionality. This is because several cloned P2X receptors from primitive species fail to form functional ATP-activated channels (Fountain et al., 2008; Ludlow et al., 2009). This review will focus on the P2X receptor homologues cloned from invertebrate and primitive non-vertebrate organisms that have been shown definitively to be ATP activated ion channels through experimentation.

## CLONED NON-VERTEBRATE AND PRIMITIVE P2X RECEPTORS

The pharmacological properties of cloned P2X receptors are summarized in **Tables 1** and **2**.

**Table 1:** Agonist sensitivity of cloned P2X receptors.

	ATP	BzATP	αβmeATP	βγimidoATP
*Ot*P2X	250	insensitive (1 mM)	> 5mM	insensitive (1 mM)
*Dd*P2X_A_	97	(3 mM, 25%)	95	15
*Dd*P2X_B_	266	ND	ND	85
*Dd*P2X_E_	511	ND	ND	(3 mM, 22%)
*Hd*P2X	45	12 (65%)	(100 μM, 50%)	ND
*Bm*P2X	70	ND	ND	ND
*Sch*P2X	22	4 (75%)	ND	ND
*Lym*P2X	6	2 (33%)	(100 μM, 37%)	ND

*
Values are given as approximates of reported EC_50_ concentrations. EC_50_ values are given as μM. ND indicates antagonist sensitivity not determined. Maximum responses for partial agonist are given in parenthesis as % maximum ATP response. Where EC_50_ values have not been determined experimentally maximum concentrations tested are given in parenthesis with response as % maximum ATP response.
*

**Table 2:** Antagonist sensitivity of cloned P2X receptors.

	Suramin	PPADS	TNP-ATP	Cu^2+^	Zn^2+^
*Ot*P2X	No block (100 μM)	No block (100 μM)	No block (100 μM)	No block (100 μM)	ND
*Dd*P2X_A_	No block (100 μM)	No block (100 μM)	No block (100 μM)	0.04	ND
*Dd*P2X_B_	No block (100 μM)	No block (100 μM)	ND	(100 nM, 85% block)	ND
*Dd*P2X_D_	ND	ND	ND	(100 nM, 30% block)	ND
*Dd*P2X_E_	No block (100 μM)	No block (100 μM)	ND	(100 nM, 70% block)	ND
*Hd*P2X	23	15	ND	20	63
*Bm*P2X	5 (partial > 300 μM)	ND	ND	ND	ND
*Sch*P2X	10	0.5 (partial > 100 μM)	ND	ND	ND
*Lym*P2X	27 (partial > 300 μM)	8	ND	(1 mM, 63%)	(1 mM, 66%)

*Values are given as approximates of reported IC_50_ concentrations. IC_50_ values are given as μM. ND indicates antagonist sensitivity not determined. % block is given where an IC_50_ value has not been determined.*

### *Schistosoma mansoni* (TREMATODE)

The first non-vertebrate P2X receptor was cloned from the human pathogen *S. mansoni*. *Schistosoma* are parasitic blood fluke and are trematodes belonging to the platyhelminthe genus. *S. mansoni* infection in humans causes schistodomiasis, a chronic illness which can lead to severe damage of multiple organs. *S. mansoni* encodes a protein that has 26–37% sequence homology with human P2X_1_–P2X_7_. Expression in *Xenopus oocytes* produces an ATP activated ion channel (*Sch*P2X) that responds to ATP with an EC_50_ of 22 μM (Agboh et al., 2004). BzATP, a full agonist at mammalian P2X_7_ receptors, acts as a partial agonist at *Sch*P2X evoking a maximal response 75% that of ATP. Though less efficacious BzATP is more potent than ATP, evoking half-maximal responses at 4 μM. ATP evoked responses at *Sch*P2X are inhibited by classical purinergic receptor antagonists PPADS and suramin with IC_50_ values of 4 and 10 μM, respectively. Permeability studies in HEK293 cells expressing *Sch*P2X reveal a high permeability to calcium (*P*_Ca_/*P*_Na_ = 3.8) that is comparable to mammalian P2X receptors. Cation substitution experiments reveal *Sch*P2X and mammalian P2X receptors have conserved ionic pore diameters. Praziquantel is a drug used in the treatment of schistodomiasis. Though the action of praziquantel is dependent upon affecting calcium homeostasis in worms (Kohn et al., 2001), praziquantel does not inhibit *Sch*P2X (Agboh et al., 2004).

### *Dictyostelium discoideum* (AMOEBA)

*Dictyostelium discoideum*, a soil-living amoeba, transitions from a community of unicellular amoebae into a multicellular fruiting body during its developmental lifecycle. This eukaryote belongs to the phylum Mycetozoa, emerging after plants and a common ancestor to fungi and animals. *Dictyostelium* amoeba display many animal cells traits including chemotaxis and phagocytosis. The chemical stimuli that govern the social behavior and complex development of *Dictyostelium* remain a subject of intense study. Prior to the discovery of ATP activated P2X receptors in *Dictyostelium* it was known that the neurotransmitters glutamate and GABA are important signaling cues governing cell fate and development in the organism (Fountain, 2010). The genome of *Dictyostelium* encodes five P2X receptor homologues (*Dd*P2X_A__-__E_) that display low primary sequence homology with human P2X receptors. The family of *Dictyostelium* P2X receptors are the most extensively studied of the primitive P2X receptor paralogues in terms of biophysics, structure-function and physiology. *Dd*P2X_A_, *Dd*P2X_B_, and *Dd*P2X_E_ form functional ATP-activated ion channels when expressed in HEK293 cells or *Xenopus* oocytes (Fountain et al., 2007; Ludlow et al., 2009; Baines et al., 2013). ATP evoked currents can be detected in HEK293 cells expressing *Dd*P2X_D_ under experimental conditions of low extracellular Na^+^ (Baines et al., 2013), but *Dd*P2X_C_ expression fails to produce functional ion channels in either HEK293 or *Xenopus* oocytes (Ludlow et al., 2009; Baines et al., 2013). ATP is a full agonist at *Dictyostelium* P2X receptors with EC_50_ values in the range 100–500 μM. *Dd*P2X_A_ receptors are most sensitive to ATP and *Dd*P2X_E_ receptors least sensitive (Fountain et al., 2007; Ludlow et al., 2009; Baines et al., 2013). αβme-ATP is a full agonist of equal potency to ATP at P2X_A_ and BzATP act as a weak partial agonist (Fountain et al., 2007). Interestingly the hydrolysis resistant ATP analog βγimido-ATP is a full agonist at *Dd*PX_A_ and *Dd*P2X_B_ receptors with 3–10-fold greater potency than ATP (Fountain et al., 2007; Ludlow et al., 2009). βγimido-ATP was found to act as a very weak agonist at *DdP2X*_E_ receptors. *Dictyostelium* P2X receptors are freely permeable to Na^+^, Ca^2^^+^ (*P*_Ca_/*P*_Na_ = 1.5; Fountain et al., 2007), K^+^ (*P*_K_/*P*_Ca_ = 1.8–2.0; Ludlow et al., 2009), NH_4_^+^ (*P*_NH4_/*P*_Ca_ = 1.8–2.0; Ludlow et al., 2009) and choline (*P*_K_/*P*_Ca_ = 0.5–0.6; Fountain et al., 2007; Ludlow et al., 2009). In addition to a range of cations, *Dd*P2X_A_ and *Dd*P2X_B_ also freely permeate Cl^-^ when expressed in *Xenopus* oocytes (Ludlow et al., 2009). Permeability to Cl^-^ is an usual feature amongst P2X receptors, however, not unique. Chick and human P2X_5_ receptors are also reported to permeate Cl^-^ (Ruppelt et al., 1999; Bo et al., 2003). Unlike mammalian P2X all *Dictyostelium* P2X receptors are insensitive to antagonism by suramin, PPADS or TNP-ATP (Fountain et al., 2007; Ludlow et al., 2009). This makes *Dictyostelium* P2X receptors very useful tools for understanding antagonist action at P2X receptors as the structural determinants of drug binding in P2X receptors are poorly defined. A common feature shared by both *Dictyostelium* and mammalian P2X receptors is modulation by divalent metal ions (Virginio et al., 1997; Coddou et al., 2003). Cu^2^^+^ potently blocks *Dd*P2X_A_ with a half-maximal inhibitory of 40 nM (Fountain et al., 2007). *Dd*P2X_B_, *Dd*P2X_D_ and *Dd*P2X_E_ are blocked to a varying degree (30–85%) by 100 nM Cu^2^^+^. Ni^2^^+^ is less potent at blocking *Dd*P2X_A_ currents (IC_50_ 60 μM; Fountain et al., 2007).

The most striking feature of *Dictyostelium* P2X receptor functionality is their exclusive intracellular residence. Although some mammalian P2X receptors exist between intracellular compartments (Qureshi et al., 2007) and the plasma membrane *Dictyostelium* P2X receptors are targeted inside the cell. Several reports confirm that *Dictyostelium* P2X receptors reside on the contractile vacuole (Fountain et al., 2007; Ludlow et al., 2009), an osmoregulatory organelle and acidic calcium store (Heuser et al., 1993; Malchow et al., 2006; Sivaramakrishnan and Fountain, 2012). In a study by Fountain et al. (2007) *Dictyostelium* cells lacking *Dd*P2X_A_ through genetic disruption were found to swell in response to hypotonic stress but lack any regulatory cell volume decrease, suggesting a severe impairment of contractile vacuole function. The phenotype was reconfirmed in a later study by Baines et al. (2013) who demonstrated that regulatory cell volume decrease could be rescued, or partially rescued, in *Dd*P2X_A_ knockout cells by overexpression of *Dd*P2X_A_, *Dd*P2X_B_, *Dd*P2X_D_, or *Dd*P2X_E_, but not *Dd*P2X_C_ which fail to form functional ion channels when expressed in HEK293 or *Xenopus oocytes* (Ludlow et al., 2009; Baines et al., 2013). These data indicate a requirement for ATP activation of P2X receptors for normal contractile vacuole function and osmoregulation. These findings are not in agreement with a study by Ludlow et al. (2009) who demonstrate that *Dictyostelium* lacking all five P2X receptors still undergo regulatory cell volume decrease, despite a delay in recovery. The differences in phenotype reported can be explained by strain variance*.* In a recent side-by-side examination (Sivaramakrishnan and Fountain, 2013) of AX2 (used by Ludlow et al., 2009) and AX4 (used by Fountain et al., 2007; Baines et al., 2013) laboratory strains of *Dictyostelium*, it was found that wild-type AX2 and AX4 vary in the degree of volume recovery following hypotonic swelling and that AX2 but not AX4 can tolerate loss of *Dd*P2X_A_. Within the vacuolar membrane P2X receptors are orientated such that the ATP binding site (ectodomain) faces the vacuole lumen, suggesting that the P2X receptors are positioned to sense changes in luminal ATP. Experiments using purified vacuoles demonstrate that ATP can be translocated into the vacuole lumen, representing a possible mechanism of ATP accumulation (Sivaramakrishnan and Fountain, 2013). Addition of ATP to intact vacuole preparations causes release of stored calcium. The magnitude of calcium release is reduced in *Dd*P2X_A_ knockout vacuoles and ablated in vacuoles lacking all five P2X receptors. These data suggest that vacuoles respond to luminal ATP accumulation by releasing stored calcium via intracellular P2X receptor activation (Sivaramakrishnan and Fountain, 2013). It remains unclear how P2X receptor dependent calcium release contributes to contractile vacuole function though possibilities include facilitation of docking or vacuole fusion. Vesicular P2X_4_ receptor activation has been shown recently to facilitate vesicle fusion in mammalian cells in a calcium-dependent fashion (Miklavc et al., 2011; Thompson et al., 2013). Vacuoles isolated from AX2 amoeba release substantially less calcium in response to ATP in comparison to AX4 vacuoles, which may provide some mechanistic insight into the difference in P2X receptor dependency for osmoregulation between the two strains (Sivaramakrishnan and Fountain, 2013).

Extracellular ATP is detectable in suspensions of *Dictyostelium* (Parish and Weibel, 1980). Early work demonstrated that application of extracellular ATP stimulates Ca^2^^+^ influx in *Dictyostelium*which was sensitive to the purinergic receptor antagonist suramin (Parish and Weibel, 1980). More recently this has been demonstrated using aqueorin expressing strains of *Dictyostelium* (Ludlow et al., 2008), though in this study the ATP evoked Ca^2^^+^ responses were insensitive to the P2 receptor antagonists suramin and PPADS but did display sensitivity to block by low micromolar Cu^2^^+^ as for the *Dictyostelium* P2X_A_ receptor (Fountain et al., 2007; Ludlow et al., 2009). Despite this, the ATP evoked calcium response remains intact following genetic knockout of all five P2X receptors (P2X_A__-__E_; Ludlow et al., 2009) suggesting the *Dictyostelium* P2X receptors do not mediate responses to extracellular ATP and supports their intracellular residency (Fountain et al., 2007; Ludlow et al., 2009; Sivaramakrishnan and Fountain, 2012).

### *Ostreococcus tauri* (ALGAE)

*Ostreococcus* are primitive single celled algae and the smallest free-living eukaryotes. They belong to the *Prasinophyceae* class of unicellular green algae that mainly includes marine planktonic species, and are close to the evolutionary origins of photosynthesis. *O. tauri* encodes a protein of 387 amino acids termed *Ot*P2X that shares 23% primary sequence identity with the *Dictyostelium* P2X_A_ receptor (Fountain et al., 2007) and around 28% identity with human P2X receptors. Expression of *Ot*P2X-*myc* in HEK293 cells produces a 50-kDa protein (Fountain et al., 2008). The receptor contains many of the residues considering important for mammalian P2X receptor function, including conservation of ectodomain lysine residues are positions equivalent to Lys^69^ and Lys^308^ of rat P2X_2_, though overall the ectodomain is poorly conserved. The N-terminal YXTXK/R sequence is retained, however, the C-terminal YXXXK motif shown to promote membrane retention in mammalian receptors (Chaumont et al., 2004) is replaced with a YESWL sequence.

ATP evokes *Ot*P2X channel opening with a half maximal concentration around 250 μM and activation threshold of around 30 μM. Whole-cell currents display modest desensitization in the presence of ligand. Single channel analysis revealed that *Ot*P2X open channel properties are flickery in nature (Fountain et al., 2008). α,β-methylene-ATP evokes very small currents though bzATP, β,γ-imido-ATP or other nucleotides triphosphates elicit no response (Fountain et al., 2008). The antagonist profile of *Ot*P2X is similar to that of the *Dictyostelium* P2X receptors with suramin, PPADS and TNP-ATP all failing to cause block up to 100 μM. Unlike the *Dictyostelium* P2X receptor, *Ot*P2X receptor currents are unaffected by Cu^2^^+^ upto 100 μM.

In contrast to other P2X receptors, *Ot*P2X displays poor calcium permeability (*P*_Ca_/P_Na_ = 0.4). The poor permeation of calcium is a result of a major structural difference between *Ot*P2X and mammalian receptors, i.e., the absence of an aspartate residue that is highly conserved amongst other primitive and mammalian P2X receptors in the second transmembrane domain, the conserved aspartate is replaced by an asparagine residue. Asn^353^ in *Ot*P2X is equivalent to Asp^349^ in rat P2X_2_. Though a switch from an acidic to a basic moiety can clearly be tolerated at this position in *Ot*P2X, a [N353A] mutation renders the receptor non-functional (Fountain et al., 2008). *Ot*P2X[N353R] enhances the calcium permeability (*P*_Ca_/P_Na_ = 0.64; Fountain et al., 2008) but not back to the level of mammalian P2X receptors. This suggests other residues in mammalian P2X receptors also contribute to high calcium permeability (Migita et al., 2001; Samways and Egan, 2007). The pore diameter of *Ot*P2X receptors estimated from the relative permeability of a range of cations suggests a permeability cut-off of 1 nm. This broadly agrees with estimates of mammalian and *Dictyostelium* P2X receptor pore sizes (Evans et al., 1996; Fountain et al., 2007) and suggests architectural conservation of the selectivity filter between very early P2X receptor proteins and mammalian P2X receptors.

Though *Ot*P2X is clearly expressed at the plasma membrane when overexpressed in HEK293 cells, the subcellular localization of *Ot*P2X in *O. tauri* has not been confirmed. Such experiments are hampered but the lack of selective antibodies to *Ot*P2X and the size of the organism (around 1 μM in diameter) not being amenable to conventional immunocytochemical studies. However, experiments to determine whether ATP can induce calcium entry in *O. tauri* suspended in artificial sea water provide some indirect data supporting a lack of cell surface expression of P2X receptors. Though ATP does not stimulate calcium influx micromolar capsaicin, the TRPV1 channel agonist, did stimulate influx. A TRPV1 homologue is encoded by this primitive algae.

### Monosiga brevicollis (Choanoflagelate)

*Choanoflagelates* are free living unicellular and colonial flagellate eukaryotes considered the closest living relative of animal cells. Fountain et al. (2008) reported cloning of a P2X receptor homologue from *M. brevicollis*. The *Mb*P2X receptor formed functional ATP activated ion channels when expressed in HEK293, though a pharmacological and biophysical characterization has not yet been published.

### TARDIGRADE *(Hypsibius dujardini)*

*Hypsibius dujardini* belongs to the phylum Tardigrade that shares features common to nematodes and arthropods. Around 400 μM in length, these multicellular organisms inhabit moss and freshwater environments, and are capable of lowering their metabolism enough to survive desiccating environments for long periods. Bavan et al. (2009) identified a 330 bp EST from *H. dujardini* which when translated shared homology with mammalian P2X receptors. The full-length coding region translates to a 480 amino acid protein (*Hd*P2X) that shares between 36 and 38% sequence homology with human P2X_1_, P2X_3_, and P2X_4_. Phylogenic analysis suggests *Hd*P2X is an ancestor of vertebrate P2X receptors and orthologous to other non-vertebrate receptors including *Dictyostelium*, *S. Mansoni* and *O. tauri*. Expression of *Hd*P2X cRNA in *Xenopus* oocytes produces ATP (EC_50_ 45 μM) activated ion channels that mediate transient inward currents that rapidly desensitizes in the presence of ligand. Despite this rapid desensitization and in contrast to the human P2X_1_ receptor that also displays rapid desensitization during ATP application, *Hd*P2X currents recover. Current amplitude is fully recovered following 5 min agonist wash-off. *Hd*P2X can be activated by both BzATP and αβ-methylene-ATP. BzATP acts as a partial agonist at *Hd*P2X with maximal concentrations producing around 65% of ATP. Though BzATP is less efficacious it acts more potently than ATP with an EC_50_ around 12 μM. α,β-methylene-ATP is less efficacious than ATP and evokes current amplitudes that are 50% of ATP responses. BzATP potency and efficacy with regard to ATP at *Hd*P2X mirror that of BzATP properties at *Sch*P2X receptors (Agboh et al., 2004).

*Hd*P2X is blocked by the broad-spectrum purinergic receptor antagonists PPADs (IC_50_ 15 μM) and Suramin (IC_50_ 23 μM), which is in contrast to other primitive P2X receptors that are insensitive to classical antagonists. Micromolar Cu^2^^+^ and Zn^2^^+^ block *Hd*P2X. Divalent metal ions modulate mammalian P2X receptor function and the *Dictyostelium* P2X_A_ receptor. Cu^2^^+^ and Zn^2^^+^ inhibit ATP-evoked responses with an IC_50_ of 20 and 63 μM, respectively. In mammalian P2X_2_ and P2X_7_ the modulatory activity of Cu^2^^+^ is mediated through interaction with histidine residues in the ectodomain. Bavan et al. (2009) produced histidine-to-alanine mutant *Hd*P2X receptors to investigate a role in divalent metal ion action. Though *Hd*P2X inhibition by Zn^2^^+^ was unaffected by alanine substitution of ectodomain histidine residues, the effectiveness of Cu^2^^+^ block was limited in *Hd*P2X[H252A/H306A] and [H232A/H306A] double mutants suggesting a role for these residues in coordinating Cu^2^^+^ action. ATP evoked currents mediated by *Hd*2X are potentiated by the macrocyclic lactone ivermectin which also potentiates mammalian P2X_4_ and *S. mansoni* receptors.

### *Lymnaea stagnalis* (POND SNAIL)

The pond snail *Lymnaea stagnalis* has proven a useful model to study fundamental aspects of the CNS. Its relatively simple CNS contains <22,000 neurons making it amenable to study processes of associative memory and taste. ATP release in molluscan CNS has been studied in real-time (Gruenhagen et al., 2004) highlighting a potential for purinergic receptors in invertebrate CNS function. A full-length P2X receptor orthologue has been cloned from *L. stagnalis* CNS (*Lym*P2X). *Lym*P2X is 435 amino acids in length and shares 31–46% identity with human P2X_1_–P2X_7_, sharing most similarity with the human P2X_4_ receptor. *Lym*P2X expressed in *Xenopus oocytes* mediates inward currents that can be activated by ATP, BzATP and α,β-methylene-ATP. ATP is a full agonist that produces half-maximal responses at 6 μM. BzATP is 3-fold more potent (EC_50_ 2 μM) than ATP at *Lym*P2X but acts as a partial agonist, producing a maximal response 66% that of ATP maximal response. α,β-methylene-ATP acts as a weak agonist. *Lym*P2X is blocked by both PPADS and suramin. PPADS can completely block ATP evoked currents above 100 μM and has a half-maximal inhibitory concentration of 8 μM. Suramin is less effective with an IC_50_ of 27 μM and produces incomplete channel block even at a concentration of 300 μM. The suramin resistant component accounts for around 40% of maximum currant (Bavan et al., 2012). *Lym*P2X currents are not potentiated by ivermectin but are potentiated by 100 μM Cu^2^^+^ or Zn^2^^+^. The level of potentiation is between 45 and 75%. However, the effect of divalent metal ions is biphasic as 1 mM Cu^2^^+^ or Zn^2^^+^ inhibits the receptor by around 65%. The CNS of *Lymnaea* has several discernable ganglia including buccal, cerebral, pedal, pleural, left parietal, right parietal, and visceral ganglia. In situ hybridization reveals widespread expression of *Lym*P2X in neurons of all ganglia, though quantitation of *Lym*P2X mRNA transcripts reveals highest expression in neurons of pedal ganglia and the lowest levels in pleural neurons (Bavan et al., 2012). Though a physiological role of *Lym*P2X is yet to be assigned, it is highly likely that the receptor is placed to respond to ATP secreted by neurons or supporting cells of the mollusc CNS.

### *Boophilus microplus* (TICK)

The *B. microplus* tick causes detrimental effects to cattle wellbeing through blood feeding and transmission of disease. The tick P2X receptor homologue *Bm*P2X forms a functional ATP activated ion channel when expressed in *xenoupus* oocytes (Bavan et al., 2011). The 414 amino acid long receptor shares between 30 and 44% sequence identity with human receptors, sharing the most identity with human P2X_4_ and least with P2X_7_. The receptor contains many structural motifs common to mammalian P2X receptors including 10 conserved ectodomain cysteines, positive and aromatic residues implicated in ATP binding and N-terminal putative protein kinase C phosphorylation site. Currents passed by *Bm*P2X exhibit extremely slow kinetics. ATP evoked currents reach peak after almost 5 s, which is in stark contrast to the millisecond activation kinetics of mammalian P2X receptors (North, 2002). Current decay in the presence of agonist are also markedly slow. *Bm*P2X currents decay by around 10% after 20 s exposure to ATP with 50% decay occurring after prolonged (>5 min) agonist application. Despite limited current decay in the presence of agonist, rundown in peak responses is marked. Consecutive ATP applications cause a 12% reduction in peak currents. Bavan et al. (2011) identified sequences positively charged residues in the C-terminus responsible for controlling receptor desensitization. Basic residues in the receptor C-terminus also control the desensitization kinetics of human P2X receptors (Fountain and North, 2006). However, the C-terminus does not contribute to receptor rundown properties. ATP activates *Bm*P2X with an EC_50_ value of 70 μM, though adenosine, ADP or UTP (all up to 1 mM) do not evoke currents. Suramin antagonizes *Bm*P2X (IC_50_ = 5 μM) but produces an incomplete block with currents persisting up to 300 μM. Ivermectin potentiates ATP evoked currents at mammalian P2X_4_ receptors (Priel and Silberberg, 2004), *S. mansoni* P2X (*Sm*P2X) and *H. dujarini* (*Hd*P2X) receptors. Despite its broad-spectrum anti-parasitic activity ivermectin does not potentiation *Bm*P2X currents. However, currents are potentiated by amitraz, a triazapentadine compound used widely in the treatment of tick infestation in cattle. Peak currents are potentiated by 23 and 94% by 1 and 100 μM amitraz, respectively. The identification of *Bm*P2X is of major interest, not only as a target for potential new anti-parasitic drugs, but also as an example on an arthropod P2X receptor. Genomic information reveals that other arthropods including *Drosophila melanogaster*, *Apis mellifera* and *Anopheles gambiae* lack P2X receptors (Fountain and Burnstock, 2009). The existence of P2X receptors in the arthropod phylum suggests selective loss of P2X receptors in some, and likely the majority, of insect species.

### GENERAL STRUCTURAL CONSERVATION WITH MAMMALIAN RECEPTORS

Functional mammalian P2X receptors assemble as oligomers of three pore-forming subunits (Young et al., 2008; Kawate et al., 2009). This trimeric oligomeric state is unusual amongst other ligand-gated and voltage-gated ion channel families, including ionotropic glutamate and nicotinic acetylcholine receptors (Cys-loop superfamily), but shared by ASIC and intracellular TRIC channels. Our previous work demonstrates that *Dictyostelium* P2X assemble as trimers, at least when expressed as recombinant receptors in mammalian cells (Fountain et al., 2007), suggesting a conservation of trimer formation by primitive P2X receptors. Expression of *Dictyostelium* P2X receptors in Sf9 insect cells also results in strong trimer formation, and the *Dictyostelium* receptor trimers are a similar size to that of vertebrate receptor trimers (Valente et al., 2011). The *Dictyostelium* P2X receptors remain the best structurally characterized primitive P2X receptors. The low primary sequence homology with mammalian P2X receptors (Fountain et al., 2007) and significantly different 3D structure (Valente et al., 2011) make them interesting candidates for future structural studies.

Ten ectodomain cysteine residues are highly conserved in mammalian P2X receptors and interact to form five disulphide bonds. The cysteines are positioned at residues C177, C126, C132, C149, C159, C165, C217, C227, C261, and C270 based on human P2X_1_ numbering. These disulphide bonds are resolved in the zebrafish P2X4 crystal structure (Kawate et al., 2009) and influence the structure of the ATP binding pocket, channel gating properties, and trafficking of mammalian receptors (Rokic et al., 2010; Jindrichova et al., 2012). The degree of ectodomain cysteine conservation in cloned primitive P2X receptors varies greatly and shows no correlation with species phylogeny. Trematode (*S. mansoni*)** and tarigrade (*H. dujardini*) receptors retain all equivalent cysteine residues, whereas algae (*O. tauri*) and choanoflagellate (*M. brevicollis*) receptors lack C217, C227, and C117, C165 equivalents, respectively. Based on the prediction of cysteine–cysteine pairing for disulphide bond formation in the human P2X_1_ receptor (Ennion and Evans, 2002), this would predict both the *O. tauri* and *M. brevicollis* receptor lack a single ectodomain disulphide bond, though at different positions. Strikingly, the *Dictyostelium* P2X_A_ receptors lacks cysteines at all equivalent position yet is a functional ATP activated ion channel (Fountain et al., 2007), suggesting a marked difference in ectodomain tertiary structure despite an ability to bind micromolar ATP (Valente et al., 2011).

### FUTURE PERSPECTIVES AND EXPERIMENTAL ADVANTAGES

A growing wealth of genomic information for single celled and non-vertebrate species makes it highly likely that our knowledge of P2X receptor phylogeny is set to expand rapidly. Recently several putative P2X receptor sequences were reported from sea sponge (*Amphimedon queenslandica*), amoeboid holozoan (*Capsaspora owczarzaki*) and nematode (*Xiphinema index*; Cai, 2012). Interestingly the same report identifies P2X orthologues in three species of basal fungi, namely *Allomyces* *macrogynus*, *Spizellomyces punctatus,* and *Batrachochytrium dendrobatidis* (Cai and Clapham, 2012). Though the function of these newly identified P2X receptors is yet to be demonstrated experimentally, these putative receptors share many of the structural hallmarks associated with P2X receptor function (Cai, 2012). Their existence suggests some phyla initially thought to lack P2X receptors, such as nematode and fungi (Fountain and Burnstock, 2009), may contain some species that do posses ATP activated ion channels. Identification of P2X receptors in the single celled green algae species *Ostreococcus tauri* demonstrate that the existence of P2X receptors predates the origins of multicellularity, and that the evolution of these receptor class occurred more than 1 billion years ago. Similar sequences are also present in the genome of *Osteococcus lucimarinus* (Palenik et al., 2007). Though *Ot*P2X share poor primary structure homology with mammalian P2X receptors the proteins assembly to fully functional ATP activated ion channels. Elucidating the physiological role of P2X receptors in such small organisms will be technically challenging but of immense interest. *O. tauri* are photosynthetic organisms yet to date the has been no functional or genomic evidence presented for the existence of P2X receptors in higher plants. P2X receptors are notably absent from some species used extensively in neuroscience as model organisms including *Caenorhabditis elegans* and *Drosophila melanogaster* (Fountain and Burnstock, 2009). The absence of functional P2X receptors in *Drosophila* has been used as an experimental advantage for the study of neural circuits and behavior in this genetically amenable model organism. Ectopic expression of rat P2X_2_ in *Drosophila* neurons allows for channel activation by laser-stimulated uncaging of caged ATP injected into specific fly brain areas. This allows pair activation of a specific set of neurons with exposure to a second stimulus such as odor (Zemelman et al., 2003).

### SUMMARY

In summary, the phylogenetic distribution of P2X receptors is incomplete but demonstration of functional receptors in simple unicellular organisms suggests evolution of this receptor class occurred over one billion years ago. The fact that many primitive P2X receptors share very low sequence homology with mammalian P2X receptors, including absence of key motifs, yet still retain micromolar sensitivity to ATP and common permeability properties is intriguing. Some low homology receptors which lack sensitivity to common P2X receptors are likely to be useful tools in the future with which to delineate the residues that coordinate drug binding at P2X receptors. Though we have gathered much structural information from cloning and characterization of primitive P2X receptor our understanding of their cell biology and physiology is restricted, but likely to provide fundamental information about why and how the P2X receptor class of ligand-gated ion channels evolved.

## Conflict of Interest Statement

The author declares that the research was conducted in the absence of any commercial or financial relationships that could be construed as a potential conflict of interest.
